# Association Between Alpha Angle and Acetabular Index in Screening for Developmental Dysplasia of the Hip

**DOI:** 10.7759/cureus.66334

**Published:** 2024-08-06

**Authors:** Jose Roberto Acosta Gomez, German Roberto Acosta Gomez, Vianey Espinosa Martinez, Aida Rosas Torres, Maria del Carmen Garcia Ruiz

**Affiliations:** 1 Orthopaedics, Hospital General de Mexico "Dr. Eduardo Liceaga", Mexico City, MEX

**Keywords:** developmental dysplasia of the hip (ddh), acetabular index, screening, hip, dysplasia

## Abstract

Background: Developmental dysplasia of the hip (DDH) is a critical orthopedic condition ranging from minor hip joint instability to complete dislocations, demanding early intervention to avoid severe complications, especially in children. In Mexico, stringent regulations under the Norma Oficial Mexicana (NOM) mandate hip screening for pediatric patients. The primary objective of this study is to investigate the relationship between alpha angles and the acetabular index in patients at six months of age, aiming to determine whether an increase in the alpha angle correlates with a better acetabular index.

Methods: We included 120 patients who were screened for hip issues with Graf's methodology in June 2023 and September 2023 at the General Hospital of Mexico "Dr. Eduardo Liceaga" in Mexico City before three months of age and attended their follow-up appointment at six months of age, where acetabular index measurement was performed using the Carestream platform on institutional X-rays.

Results: In our statistical analysis, we obtained a statistically significant relationship between an increase in the left hip alpha angle and a decrease in the left hip acetabular index (p=0.015) although it was not significant for an acetabular index of less than 25° (p=0.055). A significant relationship was observed between the right hip acetabular index and the right hip alpha angle (p=0.017) but not significant for an acetabular index less than 25° (p=0.10).

Conclusion: Universal hip screening is crucial for the early detection of DDH. Our study emphasizes using alpha-angle measurements (>70°) as reliable indicators of normal hip health.

## Introduction

Developmental dysplasia of the hip (DDH) is a significant orthopedic condition that varies from mild instability in the hip joint to complete dislocations. Prompt and effective treatment is essential to prevent long-term complications, particularly due to its high prevalence among children [1]. According to the systematic review and meta-analysis by Pakarinen et al., the incidence of DDH is 2.3 per 1,000 births in patients without a history and 3.6 per 1,000 births in those with risk factors [2]. This condition manifests in various forms, from dysplasia to malformations, with varying incidences of dislocated and dysplastic hips, attributed to racial and regional differences as well as the diagnostic methods employed [3].

Since Graf's method for evaluating DDH was introduced in 1980 [4,5], it has had an increasing global impact. This method involves measuring two angles, alpha and beta, by first identifying specific structures in the hip, such as the femoral head, acetabulum, and the labrum. Graf's classification system categorizes hips into Type I (normal), Type IIa (mild dysplasia), Type IIb (moderate dysplasia), Type IIIa (severe dysplasia), Type IIIb (dislocatable), and Type IV (dislocated) [6]. The World Health Organization (WHO) classifies DDH as the most common congenital musculoskeletal disorder, and it is notable that 54% of patients exhibit no physical symptoms [7]. For the initial evaluation of pediatric patients, early detection methods for DDH have been implemented, including imaging studies such as ultrasound. Although initially met with skepticism, ultrasound has proven effective in improving the timely detection and treatment of DDH. Currently, in Mexico, hip screening for pediatric patients is regulated and mandatory under the Norma Oficial Mexicana (NOM). [8]. The alpha angle refers to the angle formed between the acetabular roof and the vertical cortical of the iliac in the coronal plane [9]. On the other hand, the acetabular index represents the angle between the line connecting the triradiate cartilages of both hips and the bisector of the inferomedial and superolateral edges of the acetabulum [10].

The primary objective of this study is to investigate the relationship between alpha angles and the acetabular index in patients at six months of age, aiming to determine whether an increase in the alpha angle correlates with a better acetabular index. This could enhance diagnostic accuracy and the progression of DDH during infancy. This study hypothesizes a significant relationship between alpha angles and the acetabular index in patients at six months of age, proposing that an increase in the alpha angle is associated with an improved acetabular index.

## Materials and methods

In April 2024, we retrospectively conducted a review of the clinical records for patients who underwent their initial pediatric orthopedic assessment between June 2023 and September 2023 at the General Hospital of Mexico "Dr. Eduardo Liceaga" in Mexico City. These evaluations were carried out by two pediatric orthopedic surgeons and documented in each patient's records. This assessment is crucial for the early screening of the hip to identify, treat, and prevent DDH and to comply with the stipulations of the NOM. Patients eligible for inclusion were between one month and just before three months of age, had their first hip screening consultation, had recorded alpha and beta angles via ultrasound, completed medical records, attended their six-month follow-up, had X-rays on the Carestream platform, and had accurate acetabular index measurements. Exclusion criteria were patients older than three months at initial evaluation, those who missed their six-month follow-up, lacked institutional X-rays, or had incomplete medical records. Eligibility was confirmed by thoroughly reviewing medical records and patient histories. Ethical approval was waived by the local Ethics Committee of the General Hospital of Mexico "Dr. Eduardo Liceaga" due to the retrospective nature of the study and all procedures being part of routine care.

**Figure 1:**
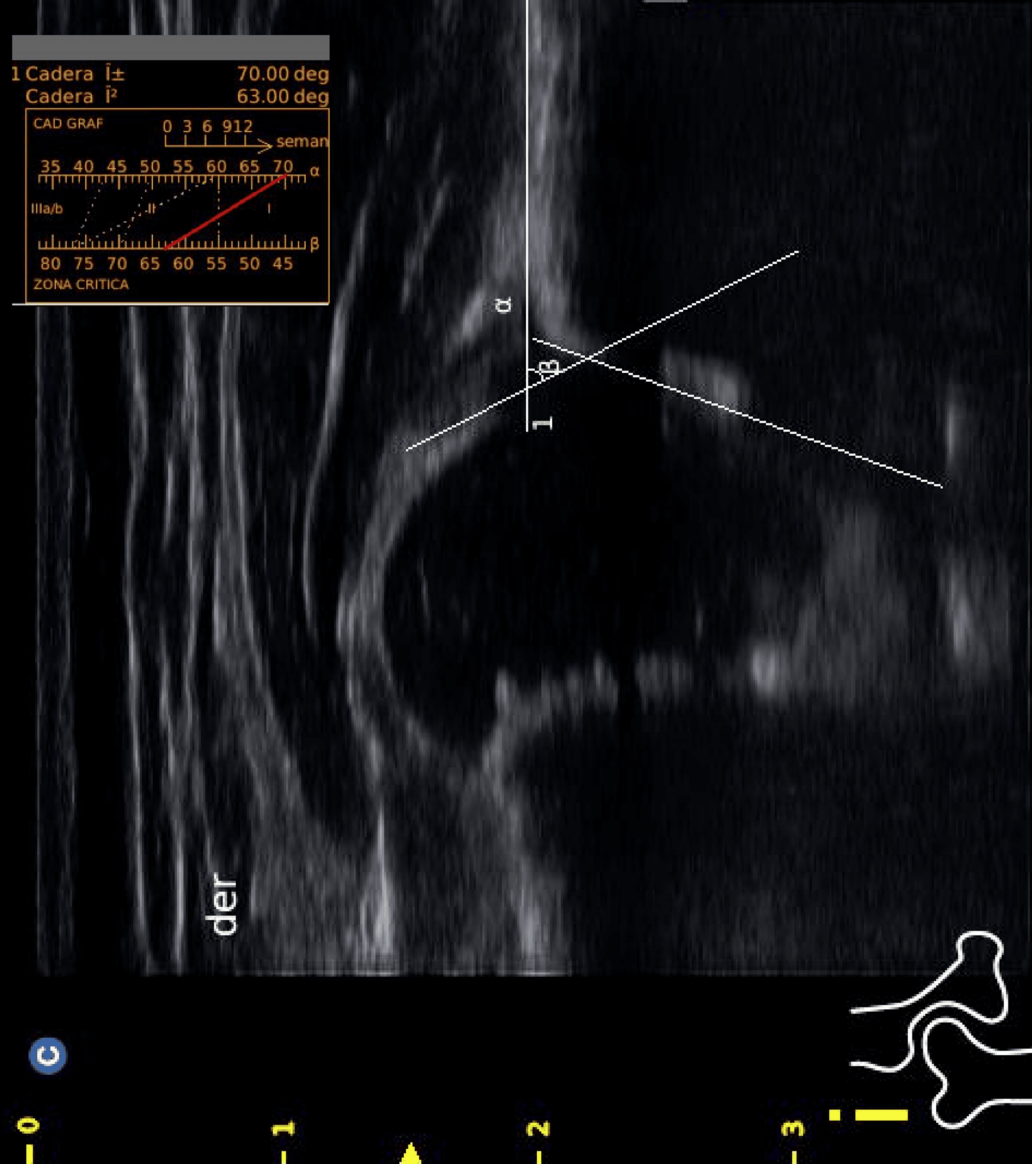
Ultrasonography of the hip: measurement of alpha and beta angles

**Figure 2:**
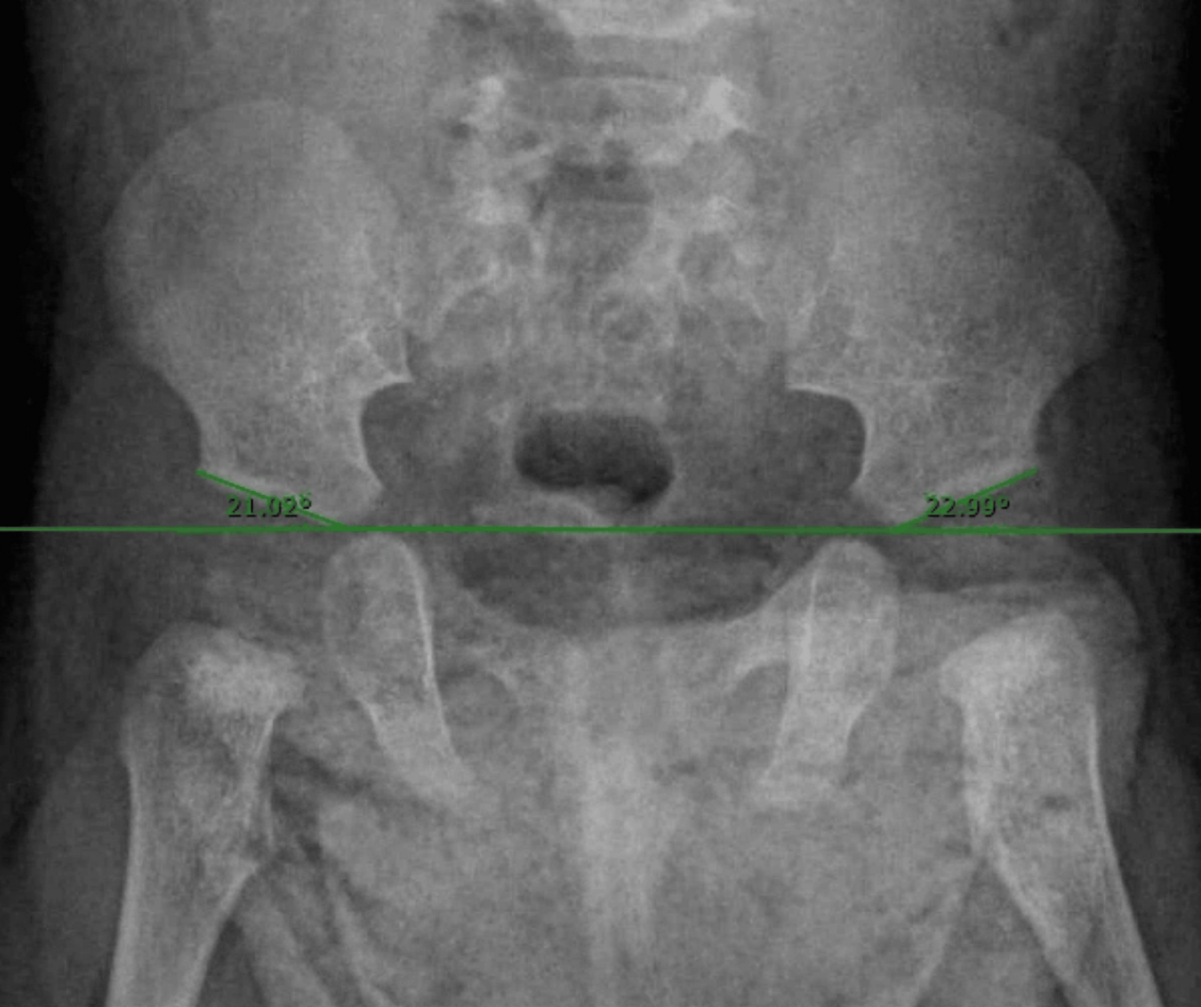
Radiographic image of the hip showing acetabular index measurements for both hips

A total of 246 patients under three months of age were included in this review. These patients completed a hip screening using Graf’s ultrasound methodology [11], where alpha and beta angle measurements were obtained. This initial screening was followed by a subsequent appointment at 6 months of age for a pelvic X-ray. At this age, the ossified femoral head was observed, and the acetabular index was measured on these X-rays. A total of 126 patients were excluded due to not attending their scheduled appointment after reaching six months of age and for not having digital X-rays available on the Carestream platform and complete medical records. A total of 120 patients who underwent hip screening before three months of age were included, with measurements of the alpha and beta angles of the right and left hips obtained via ultrasound (Figure 1). These patients attended their follow-up appointment at six months of age, during which the acetabular index of both the right and left hips was assessed. This assessment involved drawing a horizontal line through the triradiate cartilage and measuring the angle between this line and a line along the acetabular roof (Figure 2). Measurements were taken using the Carestream platform and were documented in each patient’s medical record. Both the ultrasound and X-ray measurements were performed by two pediatric orthopedic surgeons certified in Graf's method. The study seeks to find an association between the expected decrease in the acetabular index in patients with an alpha angle greater than 70°.

For our statistical analysis, we utilized the STATA/MP 14.0 platform (StataCorp LLC, College Station, TX, USA) and included the independent variables: right hip alpha angle, left hip alpha angle, right hip beta angle, left hip beta angle, right hip acetabular index, and left hip acetabular index. This study aims to find the relationship between the alpha angle measurements of the right and left hips taken during the initial screening and the radiographic measurement of the acetabular index.

## Results

The clinical records of 246 patients were initially reviewed, of which 126 were excluded for not completing the inclusion criteria. As a result, we studied a cohort of 120 patients between one and three months of age, consisting of 60 males and 60 females. Among the 60 male patients, 66.6% were delivered via cesarean, with 96.6% in a cephalic presentation and only two patients in a breech position. For the female patients, 71.6% were also born through cesarean section, and 8% of these patients had a breech presentation. We identified 76 patients with a right hip acetabular index of less than 25° as indicative of a healthy hip, of whom 61% were male and 39% were female. Additionally, 25% of patients had a right hip alpha angle greater than 70°, which is considered optimal for indicating a healthy hip. Concerning the left hip, 70 patients had a left hip acetabular index of less than 25°, with 60% being male and 40% female. Among them, 25% of patients had a left hip alpha angle greater than 70°. Regarding the Graf classification, five patients were classified as IIa for the right hip, all of whom were female. On the other hand, 10 patients were classified as IIa for the left hip, with 90% being female.

**Figure 3:**
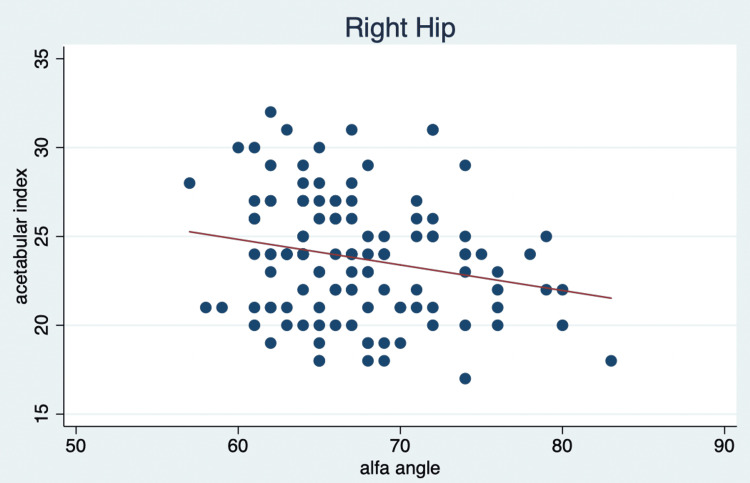
Linear regression of acetabular index and alpha angle of the right hip

**Figure 4:**
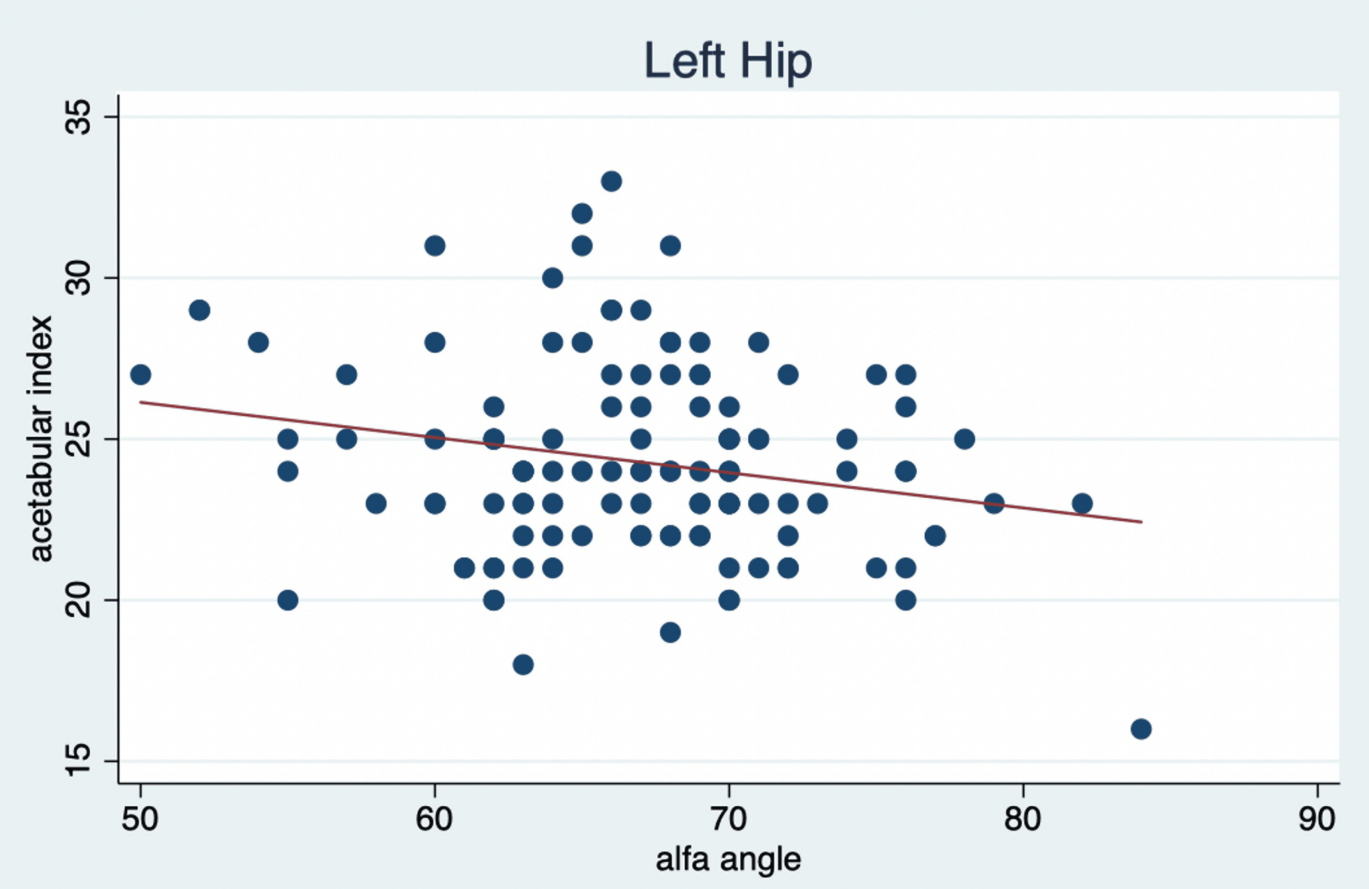
Linear regression of acetabular index and alpha angle of the left hip

From the 120 patients, we obtained a mean right hip alpha angle of 67.3° (range: 66.36-68.21%) and a mean left hip alpha angle of 66.9° (range: 65.8-68.01) at six months, with an acetabular index of 23.8° (range: 23.17-24.39) for the right hip and 24.3° (range: 23.74-24.84) for the left hip (Table 1). A Pearson correlation and linear regression analysis were conducted with the acetabular index as the dependent variable for both hips and the alpha angle as the independent variable. We identified a Pearson correlation of 0.2207 (p=0.0154) for the left hip and 0.2166 (p=0.0175) for the right hip, indicating a mild correlation. This suggests that as the alpha angle increases, the acetabular index tends to decrease slightly. Figures 3-4 reveal a notable finding: as the alpha angle of the left hip increases, the acetabular index for the left hip decreases, which is statistically significant (p=0.015), as shown in Table 2. However, this decrease in the acetabular index does not significantly relate to an index of less than 25° (p=0.059), meaning a higher alpha angle does not guarantee a healthier acetabular index below this threshold.

**Table 1:** Summary of hip data

Variable	Observations	Mean	Standard error	Range	
Right alpha angle	120	67.29167	0.4668861	66.36719	68.21615
Right beta angle	120	67.10833	0.6905521	65.74097	68.4757
Left alpha angle	120	66.90833	0.5597363	65.8	68.01667
Left beta angle	120	67.10833	0.6905521	65.74097	68.4757
Right acetabular index	120	23.78333	0.3097807	23.16994	24.39673
Left acetabular index	120	24.29167	0.2771392	23.7429	24.84043

**Table 2:** Relationship between acetabular index and alpha angle via simple linear regression

Variable	F	df	R2	p-value	95% CI	
Left alpha angle	6.04	1,118	0.0487	0.0154	0.1972877	0.0212159
Right alpha angle	5.81	1,118	0.0469	0.0175	0.2618186	0.0256511

For the right hip, there is also a significant relationship between the alpha angle and the acetabular index (p=0.017), as seen in Table 2. Yet, when examining whether the acetabular index is less than 25°, the relationship between the alpha angle and this index is not statistically significant (p=0.10) (Table 3). This indicates that while the alpha angle influences the acetabular index, it does not necessarily correspond to a healthier acetabular index of less than 25°.

**Table 3:** Summary of simple logistic regression of acetabular index less than 25° and alpha angle

Variable	Odds ratio	Standard error	Z	p-value	95% CI	
Left alpha angle	1.062059	0.0338	1.89	0.059	0.9977594	1.130502
Right alpha angle	1.06622	0.04245	1.61	0.107	0.9861901	1.152745

## Discussion

Our findings indicate that over 90% of patients with immature hips and an alpha angle below 60° were female, suggesting a potential gender predisposition. Although we found a statistically significant relationship between the alpha angle and the acetabular index, the correlation is weak. This means that while the association is statistically meaningful, a higher alpha angle does not strongly correlate with a lower acetabular index. Despite the weak correlation, the statistical significance of our results emphasizes that there is a detectable relationship between these variables.

In line with Jacobino’s research examining laterality, our study revealed that the alpha angle was more pronounced in the right hip, while the beta angle was lower, with significant statistical significance (p<0.001). However, when evaluating the Graf classification, no statistically significant differences were found between the left and right hips, consistent with the findings of our own study [12].

Other studies indicated that the incidence of DDH is notably higher in the left hip, possibly due to the fetus's habitual position during pregnancy, which tends to favor the left side [4,6,10,12,13]. This may lead to restricted abduction of the left hip during fetal development [12]. Consistent with these findings, Villanueva-Martínez et al. also reported a significant incidence on the left side, representing 47.5% compared to the right side [13]. Sari et al. observed that acetabular index values tended to be higher in women than in men, with the left hip consistently showing a greater index compared to the right [14]. In contrast, our study revealed that 66% of hips with an alpha angle below 60° were on the left side.

Current research has indicated a progressive increase in the alpha angle during the first four months of life [15]. Therefore, a significant change at six months is not expected in immature hips with an alpha angle greater than 60°. Furthermore, more recent studies, such as the one conducted by Roovers et al., indicated that 99.6% of hips classified as type I according to the Graf classification at one month of age will remain in that classification at three months of age [16]. On the other hand, Gunay has mentioned that patients with an alpha angle greater than 60° at the time of screening have femoral head coverage greater than 51% [17].

Findings obtained through ultrasound in the first three months of life are closely related to the diagnosis of DDH when evaluating acetabular index measurement at one year of age. Although this correlation is significant, cases of DDH are still identified in one-year-old children that were not detected during initial ultrasound evaluations [18].

We discovered a notable correlation between the alpha angle and acetabular index, highlighting their effectiveness in evaluating hip joint health. To strengthen these results, it's crucial to conduct thorough studies with larger participant pools and extended follow-up periods for further validation. Future studies will enhance diagnostic criteria and patient outcomes by incorporating diverse demographics and longer follow-up periods. This ongoing research is key to refining protocols, understanding DDH progression, and improving clinical strategies.

Our study presents several limitations. These include the sample size and regional focus, the cross-sectional design, measurement variability, and the lack of standardization in age. Additionally, the absence of follow-up consultations by the parents and the limited follow-up to only six months are significant concerns. Lastly, the lack of standardization in diagnostic criteria and potential biases in patient selection may also affect the results. These limitations could impact the generalizability of our findings and our ability to draw definitive conclusions. Future research should include larger sample sizes, more comprehensive follow-up, and multicenter studies that address regional and racial differences for a more thorough interpretation of the results.

## Conclusions

Universal hip screening is crucial for the early detection of DDH. Our study emphasizes using alpha-angle measurements (>70°) as reliable indicators of normal hip health, highlighting the importance of accurate diagnostics in DDH management.

The research is focused on the relationship between the alpha angle and the acetabular index in DDH screening. Analyzing a cohort of 120 patients, we observe a weak but statistically significant correlation between the alpha angle and the acetabular index. This suggests that higher alpha angles are often associated with lower acetabular indices, which may indicate the need for less frequent follow-ups during hip screenings. The accurate measurement of an alpha angle is crucial for the evaluation of hip stability and development. Detecting abnormal angles early allows for prompt and effective intervention, which helps prevent the progression of DDH and reduces potential long-term issues. Despite the relationships found in the study, future research is needed with a larger number of patients and should involve different countries and regions. This will lead to more accurate and reliable results and support better decision-making.
